# MOVING FORWARD TOWARD DEPLOYING HEALTH INFORMATION TECHNOLOGY IN ALL US NURSING FACILITIES

**DOI:** 10.1093/geroni/igad104.0964

**Published:** 2023-12-21

**Authors:** Gregory Alexander, Christina Caraballo, Maria Moen, Cynthia Morton, Terry O’Malley

**Affiliations:** Columbia University School of Nursing, New York City, New York, United States; HIMSS, Chicago, Illinois, United States; Richardson, Texas, United States; ADVION, Washington D.C., District of Columbia, United States; Massachusetts, United States

## Abstract

The Moving Forward Coalition established a health information technology (HIT) committee to develop action plans to advance the adoption of HIT in all nursing facilities. Currently, more than 70% of nursing facilities in the US have some HIT, although the levels of utilization vary greatly. Few facilities, however, have successfully implemented the capabilities required to survive in a Value Based Payment (VBP) model which Medicare will mandate by 2030. Fewer still have HIT that collects the resident’s goals, preferences, and priorities (GPPs) efficiently and in detail despite current CMS requirements for person-centered care. To the best of our knowledge, no nursing facilities possess an HIT-enabled capability to determine whether the care provided aligns with the individual’s GPPs. In this presentation, members of the HIT Committee of the Moving Forward Coalition will discuss the rationale for adopting the following three HIT priorities to realize the target goals of the Coalition and will review progress made to date: 1) develop a benchmarking tool that enables nursing facilities to understand and plan for the HIT capabilities needed to meet upcoming regulatory requirements and for successful participation in a VBP model, 2) create a taxonomy of GPPs and an HIT-based process to collect them, and 3) develop and test an HIT-enabled process to measure the concordance of care provided with an individual’s GPPs. Although the Moving Forward Coalition and the NASEM report focus on nursing facilities, the HIT tools being developed here will benefit all sites of care.

